# Efficacy of a Neurofeedback Training on Attention and Driving Performance: Physiological and Behavioral Measures

**DOI:** 10.3389/fnins.2019.00996

**Published:** 2019-09-18

**Authors:** Michela Balconi, Davide Crivelli, Laura Angioletti

**Affiliations:** Department of Psychology, Research Unit in Affective and Social Neuroscience, Catholic University of the Sacred Heart, Milan, Italy

**Keywords:** neurofeedback, driving behavior, neuropsychological measures, psychophysiological measures, DBQ

## Abstract

Increased attention and lower stress levels are associated with more functional and safe driving behavior, since they contribute to reduce distractibility and risk-taking at the wheel. Previous neuroscience research highlighted that NeuroFeedback (NF) training mediated by wearable devices could be effective in terms of neurocognitive strengthening and attention regulation with a direct effect on driving attentional performance. Thus, this research aims to test the effectiveness of a NF protocol on a sample of drivers, to observe its impact on attentional skills and psychophysiological levels of stress involved in driving behavior. 50 participants were randomly assigned to the experimental and active control group. The experimental condition consisted of a 21-day mindfulness NF training with incremental duration sessions. A pre- (*t*0) and post-treatment (*t*1) assessment included behavioral, psychometric, neuropsychological, and psychophysiological autonomic measures. Specifically, the Driver Behavior Questionnaire (DBQ) and the Active Box (AB) device were used to evaluate the everyday driving behavior. Results underlined an improvement in driving behavior performance and a decrease of violations at the wheel of the experimental group (EXPg) at *t*1 measured, respectively by AB and DBQ. About the autonomic and neuropsychological measure, an increase in heart rate (HR) and an increased accuracy at the Stroop Task were detected: a specific increase of Stroop-related HR was found for the EXPg at *t*1. Also, reduced reaction times were found in the Multiple Features Target Cancellation for the EXPg at *t*1. Overall, the EXPg displayed a physiological, behavioral and neuropsychological increased efficiency related to attention as well as a driving-related behavioral improvement after NF training.

## Introduction

Nowadays more and more research is looking for valid interventions able to improve cognitive functions required for safe driver behavior, both in the field of healthy aging, regarding older adults, and looking at young drivers. Driving behavior is a complex behavior requiring high-level cognitive functioning of attention, interference control, and low stress levels. In order to promote subjective well-being and to optimize the efficiency of neural, cognitive and behavioral reactions at the wheel – including reactions to unpredictable conditions such as weather forecasts either traffic jam – it seems important to train the ability to maintain mainly sustained attention on one’s cognitive performance and lower stress levels.

Among neurocognitive enhancement techniques, neurofeedback (NF) can be identified as a useful tool to enhance attention skills and decrease stress levels involved in driving. Recently, it has been suggested that self-enhancement may be even boosted by supporting mindfulness meditation practices with wearable, non-invasive and highly usable NF tools (Balconi et al., 2017; Sliwinski et al., 2017; Crivelli et al., 2018). Such devices can keep track of ongoing modulation of bodily and brain activity, simply by measuring physiological markers of relaxed vs. aroused or inattentive vs. focused mindsets. These technological wearable devices render meditation easier, more engaging, and rewarding, even accessible to a wider audience by providing the practitioners with real-time feedback on their engagement in practice and their related physiological underpinnings. Previous studies demonstrated the efficacy of this training on attention and perceived stress levels, as well as a relevantly decreased mental fatigue and increased vigor (Balconi et al., 2018).

Advantages in collecting multiple measurement levels of data related to driving behavior combining self-report evidence, neuropsychological tests, computerized tasks and on-road naturalistic assessment with Advanced Driver-Assistance Systems (ADAS) have been highlighted by recent works focusing on the translation of neuroscience knowledge to the driving domain (Lees et al., 2010). Moreover, we believe that the added value of a neuroscientific approach can be constituted even by the inclusion of psychophysiological measurements in the study of driving behavior. Indeed, there is an evident growing interest in evaluating physiological correlates of drivers beneath diverse stress levels and mental effort (Haigney et al., 2000; Collet et al., 2003, 2009; Healey and Picard, 2005; Mehler et al., 2010, 2012). Interestingly, two main aspects yielded to consider psychophysiological parameters as more sensitive than traditional performance measures. Firstly, an association between heart rate (HR) increase and cognitive demand or workload decrease has been reported by several previous studies (Kramer, 1991; Roscoe, 1992; Backs and Seljos, 1994; Veltman and Gaillard, 1998; Brookhuis and De Waard, 2001; Wilson, 2002). Secondly, in some contexts, such as driving performance, psychophysiological measures may be sensitive at detecting heightened cognitive load since individuals are likely to invest additional cognitive resources to maintain a given level of attentional performance as demands increase (Lenneman et al., 2005; Lenneman and Backs, 2009; Mehler et al., 2010). In line with the “doctrine of autonomic space,” Backs et al. (2005) suggested a relation between increased sympathetic activation over time and the number of executive processes involved in task performance.

More recently HR has been used to measure cognitive workload while driving (Johnson et al., 2011; Reimer et al., 2011; Mehler et al., 2012). Collectively, these studies demonstrate the feasibility of measuring HR to assess cognitive workload both in an on-road driving task and laboratory settings. However, none of the previous studies monitored on-road naturalistic driving behavior for a long period of time, neither they adopted physiological parameters as a component of a multi-measure assessment evaluating relatively stable changes before and after the experimental phase.

To date, no studies employed a NF for enhancing cognitive drivers’ performance. Resulting autonomic psychophysiological parameters recorded on-line during cognitive assessment tasks before and after such specific NF training might provide information on drivers’ stress levels. Previous pieces of evidence highlighted that an improvement in vagal tone reflected by heart rate variability (HRV) variability can be considered as a marker of global stress levels reduction (Balconi et al., 2018), and consistently, it has been suggested that HRV modulation could reflect the mediation of cortical-subcortical evaluation processes over brainstem activity and psychophysiological reactions to the context, which plays a pivotal role in guiding and adapting behavioral and stress responses (Thayer et al., 2012).

For this reason, the present study aims to test whether aspects of attention, stress management, and driving behavior can be enhanced by mindfulness-based NF practices in a sample of young healthy drivers. It is hypothesized that an experimental group (EXPg) yielding a mindfulness-based NF intervention significantly enhances performance in selective attention tasks, naturalistic driving behavior, and psychophysiological indices compared to an active control group (CNTRg). Specifically, we expected to find an increased score in the psychometric test measuring mindfulness disposition and a lower score in driving self-report aberrant behaviors for the EXPg compared to CNTRg. Better performance at the neuropsychological tests measuring attentional and executive functioning after the NF training for the EXPg was hypothesized. This improvement is supposed to be reflected even by the driving behavioral performance measured by Active Box (AB). Lastly, an increase of psychophysiological cardiovascular parameters can be hypothesized as lower stress levels after NF training for the EXPg, with reference to HRV. While an increase of HR was expected together with a boost of cognitive resources involved in driving performance after the training for the EXPg.

## Methods

### Participants

Fifty Italian subjects with valid drivers’ licenses participated in the study (38 females, 12 males; *M*_*a*__*ge*_ = 24.20, SD_*a*__*ge*_ = 6.99; *M*_*e*__*du*_ = 16.72, SD_*e*__*du*_ = 1.29). Subjects were from Northern Italy. Exclusion criteria were: a history of psychiatric or neurological diseases; the presence of cognitive deficits; ongoing concurrent therapies based on psychoactive drugs that can alter central nervous system functioning; clinically relevant stress, anxiety; the occurrence of significant stressful life events during the last 6 months; previous systematic meditation experience or analogous. Participants underwent a standardized psychometric assessment to exclude the presence of relevant clinical signs of anxiety [State-Trait Anxiety Inventory (STAI); Pedrabissi and Santinello, 1989] and of a broader set of symptoms of psychological distress [Brief Symptom Inventory (BSI); De Leo et al., 1993]. All participants were active drivers with more than 1 year of active driving experience and an annual driven distance greater than 5000 km. They all had normal or corrected-to-normal hearing and vision.

Participants were randomly divided into an experimental and an active control group. Both groups underwent mental training constituted by brief daily meditation practices. EXPg practiced with the support of wearable brain-sensing devices, while the CNTRg practiced breathing awareness. Statistical comparisons of demographic and psychometric profiles are reported in Table 1. This study was conducted according to principles of the Declaration of Helsinki and was reviewed and approved by the Ethics Committee of the Department of Psychology of the Catholic University of the Sacred Heart. All participants provided their written informed consent.

**TABLE 1:** Demographic and pre-intervention psychometric data – control and experimental group – and significance of between-group statistical comparisons.

	**Control group**	**Experimental group**	**Sig**
STAI-state	38.42(9.27)	39.00(9.43)	n.s.
BSI – global severity index	0.64(0.44)	0.67(0.50)	n.s.
BSI – positive symptoms total	20.45(9.93)	21.70(11.72)	n.s.
BSI – positive symptoms distress index	1.55(0.44)	1.51(0.40)	n.s.

*Data are shown as mean and standard deviation.*

### Procedure

**FIGURE 1:**
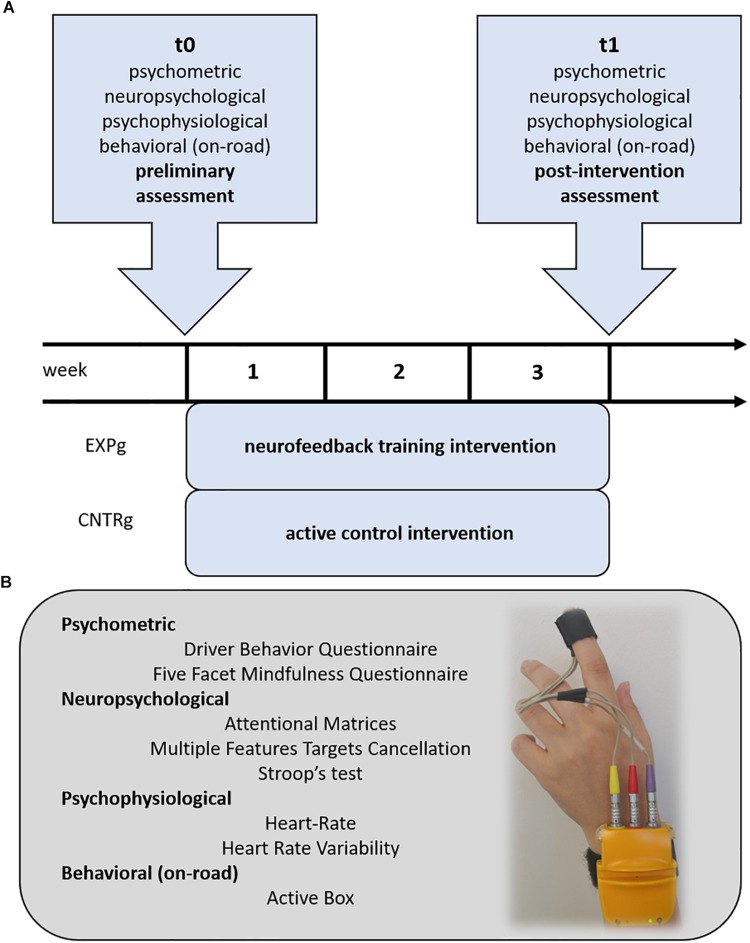
Procedure and materials. **(A)** Procedure and overall structure of the study. **(B)** List of the multi-level measures adopted in the present study. Example of Biofeedback Xpert 2000 hand montage display to measure cardiovascular response (heart rate) of participants.

The overall structure of the study included two main assessment steps – i.e., before (*t*0) and at the end of the intervention (*t*1). Psychometric and neuropsychological measures related to participants’ subjective driving behavior, dispositional mindfulness, and attention performance were collected at *t*0 and *t*1. Participants’ autonomic psychophysiological activity at rest and during a stressor task were also recorded in a quiet and darkened room at *t*0 and *t*1. In parallel with NF training intervention and active control condition, participants driving behavioral performance on-road was monitored by AB device and the dedicated app. Figures 1A,B represents the overall procedure of the study and biofeedback montage.

### Neurofeedback Intervention

The experimental intervention was based on the mindfulness approach and supported by a dedicated brain-sensing wearable device, the Lowdown Focus glasses (Smith Optics Inc., Clearfield, UT, United States). Experimental and active control interventions lasted for 21 days and included daily sessions of practice (gradually incremented duration: from 10 min a day to 20 min a day). For a depth description see Balconi et al. (2017).

### Multi-Measure Assessment

#### Psychometric Measures

The short-term effects of interventions on the driving behavior was assessed by the DBQ. In the 27 items Italian version of DBQ (Smorti and Guarnieri, 2016), respondents are required to indicate how often they do each of the violations (ordinary and aggressive violations) and mistakes (errors and lapses) when driving. Responses were recorded on a six-point scale from 0 (never) to 5 (nearly all the time).

Effects of the intervention on the dispositional mindfulness facets were measured by the Five Facets Mindfulness Questionnaire (FFMQ; Giovannini et al., 2014). The FFMQ is a 39-item instrument that measures the five aspects of mindfulness given by (1) observing, (2) describing (feelings and emotions), (3) acting with awareness, (4) non-judging of inner experience, and (5) non-reactivity to inner experience. Participants are asked to give a response on a 5-point Likert scale ranging from 1 (never true) to 5 (always true). Total score and separate scores for the five facets were computed.

#### Neuropsychological Measures

Each participant underwent a neuropsychological examination pre- and post-intervention lasting about 15 min and including the Attentional Matrices (Spinler and Tognoni, 1987), the Multiple Features Targets Cancellation (MFTC; Marra et al., 2013), and the Stroop’s test (Caffarra et al., 2002). This neuropsychological battery was administered to test visual selective attention and frontal executive functions (e.g., cognitive flexibility and sensitivity to interference) of each participant.

##### Attentional matrices

Three matrices of numbers were administered with the instruction to cross out as fast as possible target numbers of either one, two or three digits. The purpose of this test was to assess the subjects’ ability to detect visual targets among distractors. The material used in this study was the same as that in Spinler and Tognoni’s (1987) study. The overall number of targets that were crossed out within 45 s was the final score.

##### Multiple features targets cancellation

It is a visual search paper-and-pencil task, in which subjects are required to identify a target item in an array of distractors. The target consists of a square with two segments, one stemming perpendicular from the midpoint of the base, and the other stemming from the left upper corner with a 45° angle. The 67 distractors have different orientation or origins of the two lines. Two scores were obtained: time and accuracy, according to Marra et al. (2013).

##### Stroop’s test (short version)

The short Italian version of the classic Stroop’s test was used to evaluate cognitive flexibility and sensitivity to interference (Caffarra et al., 2002). Two main scores were calculated from the task: time and error interference.

#### Psychophysiological Measures

Autonomic activity and reactivity were measured at rest and during the exposure to a cognitive stressor. Resting-state recordings included alternated eyes-open and eyes-closed runs (three runs for each condition; duration: 90 s). As a cognitive stressor, we used a challenging computerized Stroop like task (Stim2 software, Compumedics Neuroscan, Charlotte, NC, United States) tapping on sustained attention and cognitive control skills. During the task, participants were presented with either congruent or incongruent color-word associations (e.g., respectively, the word “RED” written in red or the word “GREEN” written in blue) and had to discriminate between such stimuli by pressing two different buttons. We made the task stressful by manipulating time pressure and by closely presenting the randomized stimuli (stimuli duration: 300 ms; number of trials: 160). During the psychophysiological assessment, measures of cardiovascular activity were recorded via photoplethysmography by using a Biofeedback2000xpert system (Schuhfried GmbH, Mödling, Austria). For a depth description see Balconi et al. (2018). Before and after NF training HR and HRV were gathered as in Balconi et al. (2017).

#### On-Road Driving Behavioral Measures

Active Box is a device equipped with accelerometer and gyroscope with rechargeable battery and sensors which allows data relating to driving style and vehicle use to be collected. To be placed on the windshield near the rear-view mirror and connect via Bluetooth to ActiveApp. The dedicated smartphone application – ActiveApp – allows the customer to monitor his driving style thanks to a synthetic indicator (index of Performance – Overall score) attributed on the basis of some parameters, including km traveled in a year by road and time slot, respect for speed, acceleration, and deceleration limits. Each of these parameters can provide a descriptive index of distance traveled (Distance index), of km over the speed limit (SpeedLimit index), of time slot driving (TimeBand index), of accelerations and decelerations (AccDec index). The Overall score corresponds to a compound index of Performance based on the four parameters previously described. Device, app, and calculation algorithms have been designed and implemented by Infomobility.it S.p.A. This device was given to all participants and they were instructed to install it on their vehicle and to download and synchronize the dedicated app.

### Data Analysis

Psychometric, neuropsychological, psychophysiological and behavioral data were collected before and at the end of the interventions. For statistical analyses, a set of paired *t*-tests (IBM SPSS 25) using Time (*t*0 vs. *t*1) as fixed factor for both two groups (EXPg, CNTRg) was applied to mean values of psychometric, neuropsychological and psychophysiological tests collected before and at the end of the interventions. Moreover, the same paired *t*-tests using Time as fixed factor for both two groups were applied to on-road driving behavioral indices collected with AB. Preliminary Levene’s tests were computed to test homogeneity of variances between the two times and to adapt the computation of subsequent inferential tests accordingly. Potential biases related to gender were checked for and excluded. No statistically significant main and interaction effect including gender were observed; then such variable was not included in below-reported analyses.

## Results

### Psychometric Measures

**FIGURE 2:**
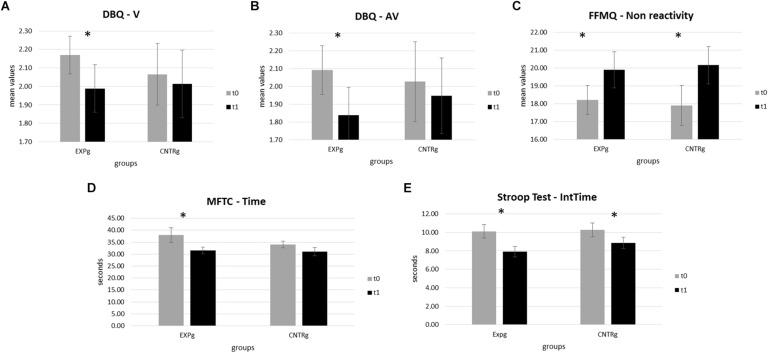
Psychometric and neuropsychological measures significant results. **(A)** Mean values of DBQ Violations for both groups at *t*0 and at *t*1. **(B)** Mean values of DBQ Aggressive Violations for both groups at *t*0 and at *t*1. **(C)** Mean values of FFMQ Non-reactivity subscale scores for both groups at *t*0 and at *t*1. **(D)** Mean values of seconds for MFCT Time performance for both groups at *t*0 and at *t*1. **(E)** Mean values of seconds for Stroop’s Test performance for both groups at *t*0 and at *t*1.

For the EXPg, statistical analysis of mean values related to whole violations at the DBQ highlighted a significant difference between *t*0 and *t*1 scores (*t*(28) = 2.136, *p* = 0.042). Specifically, violations in the EXPg at the end of the training was significantly more reduced than at the beginning (*M*_*t*__0_ = 2.2, SD*_*t*_*_0_ = 0.54; *M*_*t*__1_ = 1.98, SD*_*t*_*_1_ = 0.69). Moreover, the analysis of the mean values of aggressive violations showed significant differences between *t*0 and *t*1 for the EXPg (*t*(28) = 2.525, *p* = 0.018) with a reduction of this parameter at *t*1 compared to *t*0 (*M*_*t*__0_ = 2.09, SD*_*t*_*_0_ = 0.74; *M*_*t*__1_ = 1.84, SD*_*t*_*_1_ = 0.84). No other significant differences were found for DBQ scales for the EXPg and CNTRg (all *p* > 0.050) (Figure 2).

Statistical analysis concerning FFMQ revealed significant differences in the non-reactivity subscale only for both EXPg (*t*(28) = -2.038, *p* = 0.051) and CNTRg (*t*(28) = -2.726, *p* = 0.014) with increased scores at *t*1 compared to *t*0 for respectively EXPg (*M*_*t*__0_ = 18.21, SD*_*t*_*_0_ = 4.36; *M*_*t*__1_ = 19.91, SD*_*t*_*_1_ = 5.41) and CNTRg (*M*_*t*__0_ = 17.89, SD*_*t*_*_0_ = 4.91; *M*_*t*__1_ = 20.15, SD*_*t*_*_1_ = 4.58). No significant differences were found for FFMQ total and the other subscales scores.

### Neuropsychological Measures

Concerning MFTC neuropsychological test evaluating selective attention skills, statistical analyses highlighted a significant difference in reaction times between *t*0 and *t*1 for the EXPg (*t*(28) = 2.147, *p* = 0.041). Specifically, reaction times were reduced at the end of the training for the EXPg (*M*_*t*__0_ = 37.92, SD*_*t*_*_0_ = 16.15; *M*_*t*__1_ = 31.55, SD*_*t*_*_1_ = 7.32) compared to CNTRg (all *p* > 0.050). No differences were found for the CNTRg.

Regarding Stroop Test performance, both EXPg and CNTRg showed a similar performance. Statistical analyses highlighted a significant difference in time interference between *t*1 and *t*0 for the EXPg (*t*(28) = 3.930, *p* = 0.001) and for the CNTRg (*t*(28) = 2.170, *p* = 0.043). Specifically, time interference was reduced after training for EXPg (*M*_*t*__0_ = 10.11, SD*_*t*_*_0_ = 3.85; *M*_*t*__1_ = 7.89, SD*_*t*_*_1_ = 3.04) and the CNTRg (*M*_*t*__0_ = 10.27, SD*_*t*_*_0_ = 3.36; *M*_*t*__1_ = 8.85, SD*_*t*_*_1_ = 2.77).

No significant differences were found for error interference for both groups between *t*0 and *t*1.

No other significant differences were found for neuropsychological measures between *t*0 and *t*1 for the EXPg and CNTRg (all *p* > 0.050).

### Psychophysiological Measures

**FIGURE 3:**
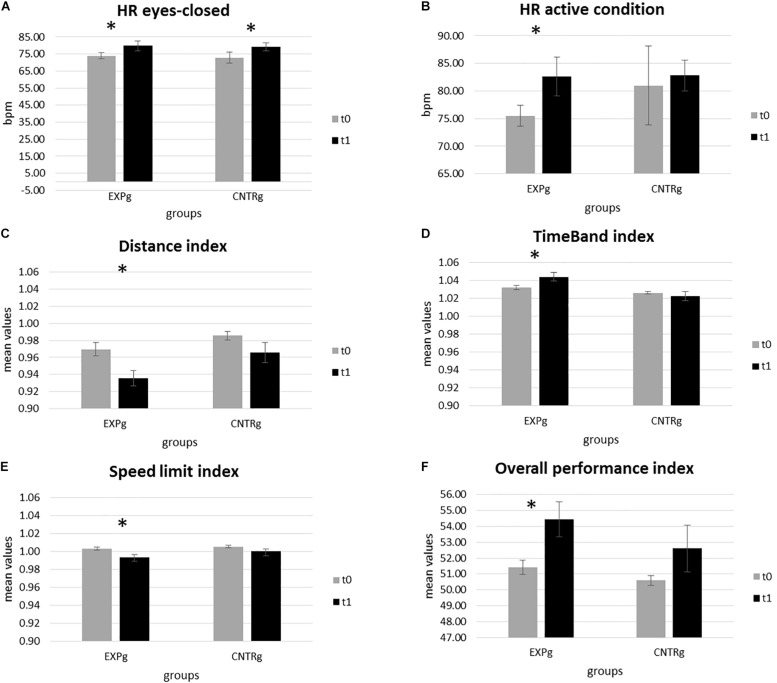
Psychophysiological and behavioral on-road driving performance (Active Box). **(A)** Significant differences in HR for eyes-closed condition for both groups at *t*0 and *t*1. **(B)** Significant differences in HR active condition for both groups at *t*0 and *t*1. Differences in for both groups before and after the experimental phase for the following indices: **(C)** Distance, **(D)** Time Band, **(E)** Speed Limit, and **(F)** Overall performance.

Concerning the analyses of HR data, the within-group difference of HR modulation proved to be significant during the eyes-closed state condition (*t*(28) = −2.061, *p* = 0.051) and during the exposure to the stressor task condition (*t*(28) = −2.050, *p* = 0.052) for the EXPg. In both cases, HR modulation was significantly greater at *t*1 than at *t*0 (eyes-closed resting-state: *M*_*t*__0_ = 73.98, SD*_*t*_*_0_ = 8.06; *M*_*t*__1_ = 79.87, SD*_*t*_*_1_ = 13.80; stressor task: *M*_*t*__0_ = 75.51, SD*_*t*_*_0_ = 9.17; *M*_*t*__1_ = 82.66, SD*_*t*_*_1_ = 16.84). For the CNTRg, HR modulation showed to be significant different between *t*0 and *t*1 only during the eyes-closed resting-state (*t*(28) = −2.280, *p* = 0.036), displaying an increase at *t*1 (*M*_*t*__0_ = 72.78, SD*_*t*_*_0_ = 14.02; *M*_*t*__1_ = 79.19, SD*_*t*_*_1_ = 9.46) similarly to the EXPg (Figure 3).

In contrast, the analysis of intervention-related HR modulation did not highlight significant differences concerning the eyes-open resting-state recording for the EXPg and CNTRg.

Statistical analyses concerning the modulation of HRV during the resting-state and the cognitive stressor task did not show significant differences between groups and times (all *p* > 0.050).

### On-Road Driving Behavioral Measures

Statistical analyses concerning AB indices revealed a significant difference between *t*0 and *t*1 in the EXPg for following descriptive indices: Distance Index (*t*(28) = 7.237, *p* < 0.001), TimeBand index (*t*(28) = −2.848, *p* = 0.019) and SpeedLimit index (*t*(28) = 4.132, *p* = 0.003).

Specifically, at the end of the training a significant reduced score was found in the EXPg for both Distance Index (*M*_*t*__0_ = 0.97, SD*_*t*_*_0_ = 0.02; *M*_*t*__1_ = 0.94, SD*_*t*_*_1_ = 0.03) and SpeedLimit index (*M*_*t*__0_ = 1.00, SD*_*t*_*_0_ = 0.01; *M*_*t*__1_ = 0.99, SD*_*t*_*_1_ = 0.01); while TimeBand index was significantly increased in *t*1 compared to *t*0 (*M*_*t*__0_ = 1.03, SD*_*t*_*_0_ = 0.01; *M*_*t*__1_ = 1.04, SD*_*t*_*_1_ = 0.01).

In addition, statistical analyses on the Overall score highlighted a significant increase of values from *t*0 to *t*1 in the EXPg (*t*(28) = −3.728, *p* = 0.005). Indeed, at the end of the training EXPg displayed increased merit scores (*M*_*t*__0_ = 51.42, SD*_*t*_*_0_ = 1.39; *M*_*t*__1_ = 54.44, SD*_*t*_*_1_ = 3.45).

No other significant differences were found for AB indices between *t*0 and *t*1 for the EXPg and CNTRg (all *p* > 0.050).

## Discussion

The present study analyzed the effects of a mindfulness-based NF training with the support of wearable brain-sensing device on the improvement of attentional performance, reduction of stress levels and behavioral on-road naturalistic driving performance in healthy young drivers. The modulation of several variables derived from a multi-measure assessment (self-report, neuropsychological, psychophysiological and behavioral level) administered before and after the interventions (experimental and active control) was considered. Statistical analyses displayed three main interesting findings related to attentional performance, stress levels and behavioral performance while driving on-road.

Firstly, a positive effect on attentional performance was revealed mainly by neuropsychological and psychophysiological data. Indeed, a decrease in reaction times for MFTC for the EXPg could support the improvement in attentional visual skills in a condition where a serial scan of stimuli is necessary to identify a specific perceptual target (Marra et al., 2013). This effect can be confirmed even by a decrease in reaction times at the Stroop’s test after the training for the EXPg that is in line with previous studies adopting NF technique supported by wearable devices (Bhayee et al., 2016). This improvement of attentional performance may be related to the data at the psychophysiological level. Indeed, in a resting state condition (eyes-closed), EXPg displayed an increase of HR, that on one side previous literature on cardiac control related to an increase of executive functions involved in a task (Backs et al., 2005; Lenneman and Backs, 2009), while on the other side literature on meditation addressed this effect at resting to the concept of a “meditation paradox” since a variety of relaxation and meditative techniques may produce active rather than quiescent cardiac dynamics, associated with increases in mean resting HR (Peng et al., 2004).

A significant effect for Stroop’s test time interference and HR at rest was also found for CNTRg. In the first case, it is possible that the paper-and-pencil Stroop’s test version (Caffarra et al., 2002) is less sensitive to discriminate changes between groups than the computerized version previously adopted in our studies (Balconi and Crivelli, 2019). Otherwise, given the nature of the active CNTRg, it might be that the intervention has partially increased the ability of subjects to control interference deriving from the environment and to focus on their performance, both when active and when at rest, even if they were only required to focus on their breath and listen to nature sounds. This second option may provide an explanation of FFMQ non-reactivity subscale result for which both groups seemed to develop the tendency to allow thoughts and feelings to come and go, impacting on stress in a non-specific way.

Secondly, a specific effect for HR during the stressor task was found for the EXPg only, thus suggesting a peculiarity and a distinct impact of the present NF protocol. Previous studies interpreted the specific increase of HR as related to the ability to face and manage the cognitive load (Lenneman et al., 2005; Lenneman and Backs, 2009; Mehler et al., 2010). Thus, it is possible that after NF training the EXPg was able to functionally employ high-level cognitive functions during task performance, and that this greater cognitive investment was modulated by an increased sympathetic activation. Indeed, differently from what expected and showed by previous studies, no differences were found for HRV (Balconi et al., 2018). Findings at the psychophysiological level could have reflected the effect of NF intervention on increased cognitive activation mechanisms (Lenneman and Backs, 2009), more than stress.

Thirdly, previous effects were reinforced by drivers’ behavioral performance measured by DBQ and with AB, namely by observing changes after NF training for the EXPg. Firstly, EXPg was less prone to engage in violations, specifically aggressive violations, after the NF treatment. These two aberrant behaviors have been previously positively related to higher accident involvement in a population of taxi drivers (Vahedi et al., 2018). Also, Sani et al. (2017) suggested a relationship between attentional bias and risky driving behavior. Thus, these deviant behaviors decrease can be interpreted as an improvement in driving behavior derived from a specific increase in attentional skills after NF training, that may have a positive impact in reducing the risk of accident involvement. In line with this, despite the EXPg seems to spend more time at the wheel for shorter distances after the intervention, AB recorded a reduction of speed limit index and better overall performance. This renders our protocol a promising way to improve not only attention but also driving skills in healthy young drivers.

To conclude, the EXPg receiving the NF training displayed a better profile in terms of alertness and attentional visual skills as revealed by the MFTC, confirmed by an augmented physiological activation as revealed by HR during a stressor task. This profile was completed by a diminished self-report mean of violations and time spent over the speed limit while driving. These laboratory findings are confirmed by the naturalistic overall performance AB index, that was higher for EXPg compared to CNTRg. To apply a multi-level set of measures may allow defining the profile of a performing driver, that in our study was mainly characterized by higher attentional levels and enhanced physiological cardiovascular activation, combined with amelioration in driving behaviors in ecological contexts.

The causal relationship between stress and cognitive performance should be explored in-depth by future studies. Our NF protocol seems to have a better impact on attention skills and on driving-related behavioral outcomes. On-road naturalistic measurement is one of the big advantage of our study compared to other research who found autonomic indices as more sensitive than driving behavior assessment (Lenneman and Backs, 2009). AB implementation constitutes the added value of this study and our positive results may encourage the adoption of ADAS to estimate evident changes in driver’s performance. This protocol has three main practical relevancies. Firstly, it introduces clearly defined training to control stress dedicated to specific categories of subjects, who showed detailed responsiveness to stress conditions; secondly, it promotes neurofeedback applications to prevent stress-related effects; thirdly, it may be a solution for controlling and limiting consequences related to cognitive impairment or cognitive decline.

Despite its innovativeness, this study is not without limitations. Future studies should increase the sample size and extend the geographical origins of drivers to allow results to be generalized to the overall drivers’ population. In addition, our protocol considered only a specific age range of participants, while similar studies in the context of driving behavior tend to compare drivers with different ages. Furthermore, the choice of an active control group was made to avoid that the effect found for the EXP group could be interpreted only as consequent to an “active” condition-training, with possible misunderstanding of results, to be imputable to the general training activity of the EXP group, instead of to the real effect of training activity itself. However, further studies might consider integrating present results with a non-active control group.

Moreover, regarding the interpretation of the psycho physiological data, an augmented HR after the intervention could also be related to a higher occurrence of stress rather than an increased executive functions capability for the participants. Besides HRV did not report significant variations, so that future studies need to consider this parameter and to deepen stress variations. While HR differences in the EXP group was a marginal significant result with further confirmation needed.

In the next future, it could be interesting to apply this NF protocol to a population of older drivers in order to verify the present results across the life span and to compare this intervention to other cognitive training aimed at improving driving skills in elderly (Casutt et al., 2014). Finally, previous studies in this field demonstrated that this protocol is suitable for healthy young participants (Crivelli et al., 2018; Balconi and Crivelli, 2019), for athletes (Crivelli et al., 2019a) and for the managerial contexts (Crivelli et al., 2019b). Nevertheless, several other contexts (e.g., hospital emergency department) could benefit from this protocol able to enhance attention and moderate stress levels.

## Data Availability

The datasets generated for this study are available on request to the corresponding author.

## Ethics Statement

The studies involving human participants were reviewed and approved by the Department of Psychology, Catholic University of the Sacred Heart of Milan, Italy. The patients/participants provided their written informed consent to participate in this study.

## Author Contributions

MB, DC, and LA contributed to the conception, design of the study, manuscript revision, and read and approved the submitted version. MB and LA wrote the first draft and each sections of the manuscript.

## Conflict of Interest Statement

The authors declare that the research was conducted in the absence of any commercial or financial relationships that could be construed as a potential conflict of interest.
